# The First Isolation of Insect-Specific Alphavirus (*Agua Salud alphavirus*) in Culex (Melanoconion) Mosquitoes in the Brazilian Amazon

**DOI:** 10.3390/v16091355

**Published:** 2024-08-24

**Authors:** Bruna Ramos, Valéria Carvalho, Eliana da Silva, Maria Freitas, Landeson Junior Barros, Maissa Santos, Jamilla Augusta Pantoja, Ercília Gonçalves, Joaquim Nunes Neto, José Wilson Junior, Durval Vieira, Daniel Dias, Ana Cecília Cruz, Bruno Nunes, Sandro Silva, Carine Aragão, Alexandre Casseb, Lívia Martins

**Affiliations:** 1 Department of Arbovirology and Hemorrhagic Fevers, Evandro Chagas Institute, BR 316, Km 07, s/n, Ananindeua 67030-000, PA, Brazil; valeriacarvalho@iec.gov.br (V.C.); elianapinto@iec.gov.br (E.d.S.); maria_freitas17@hotmail.com (M.F.); landesonbarros@iec.gov.br (L.J.B.); maissasantos@iec.gov.br (M.S.); jamillapantoja@iec.gov.br (J.A.P.); erciliagoncalves@iec.gov.br (E.G.); joaquimneto@iec.gov.br (J.N.N.); josejr@iec.gov.br (J.W.J.); durvalvieira@iec.gov.br (D.V.); danieldias@iec.gov.br (D.D.); anacecilia@iec.gov.br (A.C.C.); brunonunes@iec.gov.br (B.N.); spatroca@gmail.com (S.S.); carinefaragao@gmail.com (C.A.); liviamartins@iec.gov.br (L.M.); 2 Graduate Program in Virology, Evandro Chagas Institute, BR 316, Km 07, s/n, Ananindeua 67030-000, PA, Brazil; 3 Institute of Animal Health and Production, Federal Rural University of Amazônia, President Tancredo Neves Boulevard, 2501, Belem 66077-830, PA, Brazil; alexcasseb@yahoo.com.br

**Keywords:** insect-specific viruses, *Agua Salud alphavirus*, mosquitoes, Amazon

## Abstract

Advances in diagnostic techniques coupled with ongoing environmental changes have resulted in intensified surveillance and monitoring of arbovirus circulation in the Amazon. This increased effort has resulted in increased detection of insect-specific viruses among hematophagous arthropods collected in the field. This study aimed to document the first isolation of *Agua Salud alphavirus* in mosquitoes collected within the Brazilian Amazon. Arthropods belonging to the family Culicidae were collected within a forest fragment located in the Environmental Protection Area of the metropolitan region of Belem. Subsequently, these specimens were meticulously identified to the species level. Afterward, the collected batches were macerated, and the resulting supernatant was then inoculated into C6/36 and Vero cell cultures to facilitate viral isolation. The presence of arboviruses within the inoculated cell cultures was determined through indirect immunofluorescence analysis. Furthermore, positive supernatant samples underwent nucleotide sequencing to precisely identify the viral strains present. Notably, a batch containing *Culex* (*Melanoconion*) mosquitoes was identified to be positive for the genus *Alphavirus* via indirect immunofluorescence. This study is the first report on insect-specific alphavirus isolation in Brazil and the first-ever description of *Agua Salud alphavirus* isolation within Amazon Forest remnants.

## 1. Introduction

Since the 1950s, the Brazilian Amazon has served as a focal point for studies on the circulation of viruses pertinent to One Health. During this period, over 2000 different viral strains have been unearthed, including arboviruses [1]. Notably, approximately 212 different types of arboviruses have been isolated in the Amazon, with 36 of them holding significance in medical and veterinary contexts, spanning across genera such as *Orthobunyavirus*, *Orthoflavivirus*, and *Alphavirus* [2,3].

Environmental changes triggered by unrestrained human actions, such as excessive emissions of pollutants, improper waste disposal, rampant mining, urbanization, and deforestation, directly contribute to the emergence and resurgence of viruses with major implications for One Health in the Amazon region [4]. Disorganized urbanization is one of the main causes of forest fragmentation in the Amazon. This fragmentation gives rise to forest corridors surrounded by large urban centers, bringing humans, wild animals, and vectors together. Consequently, this phenomenon amplifies the likelihood of novel disease emergence and facilitates the urban adaptation of previously wild zoonotic viruses [3,4].

Situated within the metropolitan region of Belem, Pará, Brazil, the Environmental Protection Area of the metropolitan region of Belem (APA-Belem) is an environmental conservation area, encompassing remnants of the Amazon biome [5]. Notably, approximately 30 different strains of arboviruses have been isolated from both animals and vectors inhabiting this area. Among these, alphaviruses that cause encephalitis in humans and animals, such as the *Eastern equine encephalitis virus* (EEEV), *Western equine encephalitis virus* (WEEV), *Mucambo virus* (MUCV), and *Pixuna virus* (PIXV) [2], have been discovered.

The amalgamation of technological innovations in diagnostic methodologies and the relentless flux of environmental dynamics has catalyzed an unprecedented surge in arbovirus surveillance and monitoring efforts. As a result, this concerted endeavor has led to the unearthing of new infectious agents within hematophagous arthropod specimens collected in the Amazon region. Notably, these findings have significantly contributed to the discovery of new viruses, including insect-specific viruses (ISV) [6,7,8].

Insect-specific viruses represent a unique category of RNA viruses that naturally infect insects and are distinguished by their inability to replicate in vertebrate cells both in vitro and in vivo [9,10,11]. These viruses span several viral groups, including the main families infecting plants and taxa comprising arboviruses such as the genus *Alphavirus* [10,12]. Currently, five distinct insect-specific alphaviruses (ISA) have been identified, namely, the *Eilat virus* (EILV), *Taï Forest alphavirus* (TALV), *Mwinilunga alphavirus* (MWAV), *Yada yada virus* (YYV), and *Agua Salud alphavirus* (ASALV) [13,14,15,16,17].

*Agua Salud alphavirus* was isolated for the first time in batches containing *Culex* (*Culex*) *declarator* mosquitoes collected in Panama, in 2013 [17]. The genome of ASALV is characterized by a complete sequence spanning 11,468 nucleotides (nt) and boasts two Open Reading Frames (ORF) containing a blend of conserved and non-conserved elements [17]. Phylogenetic analyses have shown a genetic relationship between ASALV and other insect-specific alphaviruses (ISA), forming a basal group akin to the pathogenic Western equine encephalitis virus group. However, similar to other ISA counterparts, ASALV shows a notable inability to replicate in vertebrate cells (in vivo and in vitro) [17].

Despite being placed under increasing scrutiny, ASALV and other ISA remain poorly understood regarding their biological and ecological properties, including their mode of transmission, natural reservoirs, and main hosts. Furthermore, to date, there are no reports on the isolation of insect-specific alphaviruses from arthropod samples collected in the Brazilian Amazon, with reports on the isolation of these viruses restricted to countries in the Middle East [13], Africa [14,15], Australia [16], and Central America [17].

This study aimed to report the first isolation of ASALV from mosquitoes of the subgenus *Culex* (*Melanoconion*) collected in a forest fragment to the Amazon biome, during a surveillance study on arbovirus circulation at the APA-Belem in 2019 to 2020.

## 2. Materials and Methods

### 2.1. Study Area

This study was conducted at the main campus of the Federal Rural University of Amazônia (UFRA), in Belem, Pará, Brazil, situated at geographic coordinates 1°27′31″ S, 48°26′04.5″ W. The location boasts a diverse landscape including discontinuous secondary forest fragments interspersed with semi-urban areas, with buildings, pastures, roads, plantations, and animal farming facilities. Spanning approximately 215.230 hectares, the campus is nestled within the confines of the APA-Belem.

### 2.2. Ethical Consent

This study was approved by the Brazilian Institute of Environment and Renewable Natural Resources (SISBIO/IBAMA), under registration number 63,488.

### 2.3. Hematophagous Arthropod Collection and Taxonomic Identification

**Figure 1:**
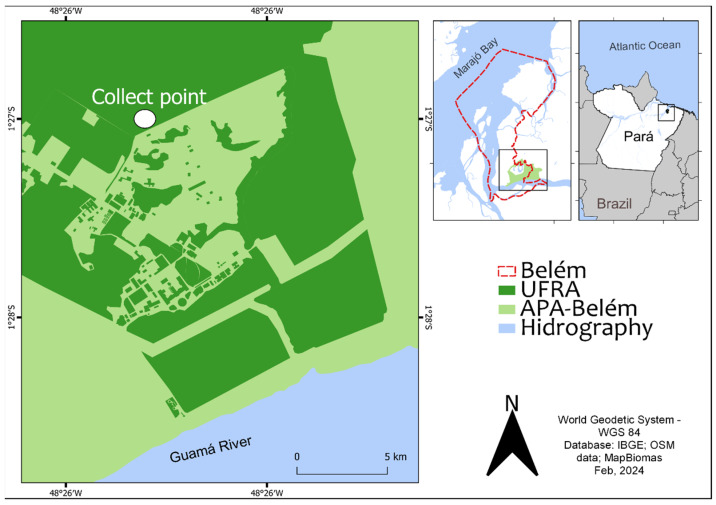
The secondary forest fragment within the APA-Belem where Culicidae mosquitoes were collected.

The collection of hematophagous arthropods placed over four ten-day expeditions within the APA-Belem, during the rainy season (February/March), rainy–dry transition season (June/July), and dry season (September/October) in 2019, followed by an additional expedition during the rainy season in March 2020. Adult insects were collected using the Protected Human Attraction technique (PHAT) [18] and *CDC Light Traps* (BioQuip Products^®^, Compton, CA, USA) deployed within a fragment of secondary forest (Figure 1). Both techniques were applied at ground level and in the tree canopy.

The taxonomic identification of the collected arthropods was conducted using dichotomous keys as proposed by Consoli and Oliveira [19]. Batches were compiled with mosquitoes of the same taxonomic classification and collected utilizing the same technique on the same date and site. This study included only insects of the family Culicidae.

### 2.4. Viral Isolation in Cell Cultures and Indirect Immunofluorescence (IIF) Test

For viral isolation, mosquitoes were ground in 1× Dulbecco’s phosphate-buffered saline (D-PBS) solution (Life Technologies, Carlsbad, CA, USA) supplemented with 5% fetal bovine serum, 2% penicillin/streptomycin, and 1% amphotericin B (Gibco, Waltham, MA, USA), using a 3 mm tungsten sphere and the TissueLyser system (Quiagen, Hilden, Germany) at a frequency of 25 Hz/1 min. Subsequently, the resulting suspensions were flash-frozen at −80 °C in a freezer for 24 h, followed by thawing and centrifugation at 10,000× *g* rpm for 10 min in a refrigerated centrifuge at 4 °C. Upon obtaining the suspensions, 150 µL of the supernatant was simultaneously inoculated in individual culture tubes (TPP^®^), each containing a monolayer of *Aedes albopictus* cells (clone C6/36; ATCC: CRL 1660) [20] and *Cercopithecus aethiops* renal cells (Vero; ATCC: CCL 81) [21].

The culture maintenance regimen involved the sustenance of C6/36 cells in Leibowitz L-15 medium modified with L-Glutamine, supplemented with penicillin (10,000 U/L), streptomycin (10,000 µg/L), 1% non-essential amino acids, 2.95% Tryptose phosphate, and 5% fetal bovine serum, and Vero cells were cultured in medium 199 supplemented with penicillin (10,000 U/L), streptomycin (10,000 µg/L), and 5% fetal bovine serum (Gibco, Waltham, MA, USA). The inoculated cultures were maintained in the incubation chamber, with the C6/36 cells maintained at 28 °C and Vero cells at 37 °C, for seven consecutive days. Throughout this incubation period, daily assessments were conducted using an inverted field microscope to check for the presence of a cytopathic effect (CPE).

On the seventh day post-inoculation (dpi), all cell cultures previously inoculated with hematophagous arthropods were collected onto slides and subjected to indirect immunofluorescence (IIF) analysis, following an adapted protocol based on the methodology proposed by Gubler et al. [22]. Polyclonal antibodies, produced in-house in Swiss albino mice (*Mus musculus*) and diluted 1:20 in PBS, were used to screen the positive samples per genus of viruses. These antibodies were specifically tailored to target arboviruses from the families *Togaviridae* (genus *Alphavirus*), *Flaviviridae* (genus *Orthoflavivirus*), *Peribunyaviridae* (genus *Orthobunyavirus*), *Phenuiviridae* (genus *Phlebovirus*), and *Sedoreoviridae* (genus *Orbivirus*) (Table 1). The immunoreaction was revealed using conjugated anti-mouse antibodies at a 1:900 ratio (Cappel, Solon, OH, USA) supplemented with Evans blue to identify negative cells. Following the completion of the test, the slides were read under a fluorescence microscope (Evos M5000, Thermo Fisher Scientific, Waltham, MA, USA).

### 2.5. Next-Generation Sequencing (NGS) and Nucleotide Sequence Assembly

Cell cultures inoculated with mosquito samples that were positive in the IIF test for the viral genus or exhibited cytopathic effects in the inoculated cultures were considered positive for virus isolation. Subsequently, to identify the viruses isolate, the samples underwent next-generation sequencing (NGS) using the NextSeq 500 platform (Illumina, Inc., San Diego, CA, USA) and the NextSeq 500/550 High Output kit v2.5 (300 cycles), following the protocol described by the manufacturer and the paired-end sequencing methodology. The generated sequences were assembled using the de novo methodology in SPAdes [23] and IDBA-UD [24] software. Posteriorly, the amino acid sequences obtained were compared to different protein databases available in the InterProScan software (https://www.ebi.ac.uk/interpro/download, accessed on 10 November 2023). The resulting contigs were tabulated, inspected, and compared with complete arbovirus sequences using Geneious software v.9.1.4 (https://geneious.com/, accessed on 10 November 2023).

### 2.6. Phylogenetic Inference

Phylogenetic inference was carried out by aligning the coding regions of viral sequences obtained in this study, which were then juxtaposed with sequences corresponding to the coding regions of the identical proteins in virus specimens cataloged in the NCBI GenBank database. Multiple sequence alignment (MSA) was executed using Maft software v.7 [25]. Phylogenetic trees were reconstructed in the IQ-TREE software v.1.6.12 [26] using the maximum likelihood (ML) method [27] and considering a bootstrap value of 1000 replicas [28]. FigTree v.1.4.4 (https//:www.github.com/rambaut/figtree/releases/tag/v.1.4.4, accessed on 10 November 2023) and InkScape v.1.1 (https//:inkscape.org/release/inkscape-1.1/, accessed on 10 November 2023) were used to graphically visualize the phylogenetic trees and produce the final images.

## 3. Results

### 3.1. Viral Isolation and Indirect Immunofluorescence Test

**Figure 2:**
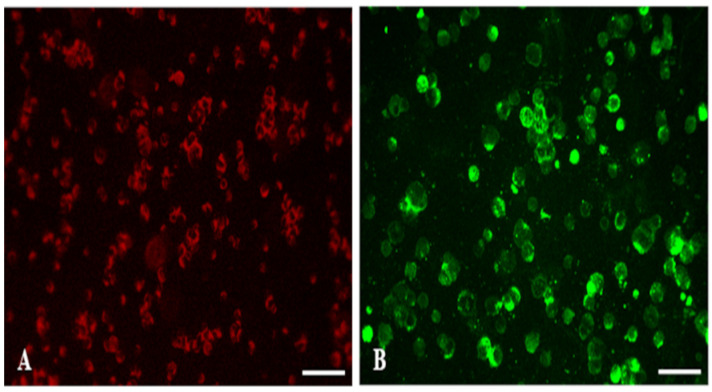
Photomicrographs of C6/36 cells inoculated with a batch of *Culex* (*Melanoconion*) mosquitoes (BE AR 867257) collected in a forest fragment at the APA-Belem showing the IIF reaction to polyclonal antibodies specific to the genus *Alphavirus*. Magnification: 200×. Scale bar: 75 µm. The cells were stained with Evans blue; the negative cells are stained in red, and the positive cells are stained in green. (**A**) C6/36 cells not infected, negative control. (**B**) C6/36 cells inoculated with the batch BE AR 867257 demonstrating a positive reaction to *Alphavirus* (4th passage, 4th dpi).

A total of 420 Culicidae batches were inoculated into C6/36 and Vero cell cultures. Among these, a batch of 41 adult female mosquitoes of the subgenus *Culex* (*Melanoconion*), collected using a *CDC Light Trap* set at ground level and identified in the laboratory as BE AR 867257 showed changes suggestive of CPE in C6/36 cells after four days post-inoculation and also displayed an IIF positive reaction for the genus *Alphavirus* (Figure 2). Conversely, Vero cells inoculated with the same batch showed no CPE during the monitoring period and were negative for the antibodies analyzed via IIF. The mentioned sample was negative for all the other groups of polyclonal antibodies tested (both cells). Since the sample BE AR 867257 showed positivity to the *Alphavirus* genus in the IIF test, we performed nucleotide sequencing analysis to identify the alphavirus responsible for the infection.

### 3.2. Nucleotide Sequencing and Phylogenetic Inference

Nucleotide sequencing analysis using the supernatant of C6/36 cells inoculated with the batch that tested positive for the genus *Alphavirus* via IIF revealed a sequence of 11,441 nt, with the 5′ and 3′ regions composed of 56 and 325 nt, respectively, and a total coding region of 10,907 nt, compatible with the complete ASALV genome available in the NCBI database, with 96.96% of amino acidic identity with structural proteins and 98.19 with non-structural proteins (Table 2 and Table 3), confirming the results obtained in the IIF test which pointed to an alphavirus present in the sample. The ASALV genome is composed of three ORFs. The ORF1 (nsP1-3) consists of 5418 nt, with a molecular weight of 200.229 kDa, and encodes non-structural proteins related to viral replication. The ORF2 (nsP4) consists of 1782 nt, with a molecular weight of 66.427 kDa, and encodes non-structural proteins. The ORF3 consists of 3747 nt, with a molecular weight of 136.388 kDa, and encodes structural proteins of the viral capsid and envelop. The sequence obtained was deposited in GenBank with identification number OQ749792.

The identity matrix comparing the degree of nucleotide and amino acid similarity between the structural proteins (Table 2) and non-structural proteins (Table 3) of the ASALV strain isolated in the APA-Belem and other insect-specific alphaviruses proteins showed a higher nucleotide and amino acid similarity between the structural and non-structural proteins of the APA-Belem isolate and the ASALV strains isolated in Panama in 2013 (MK9591114 and MK959115).

**Figure 3:**
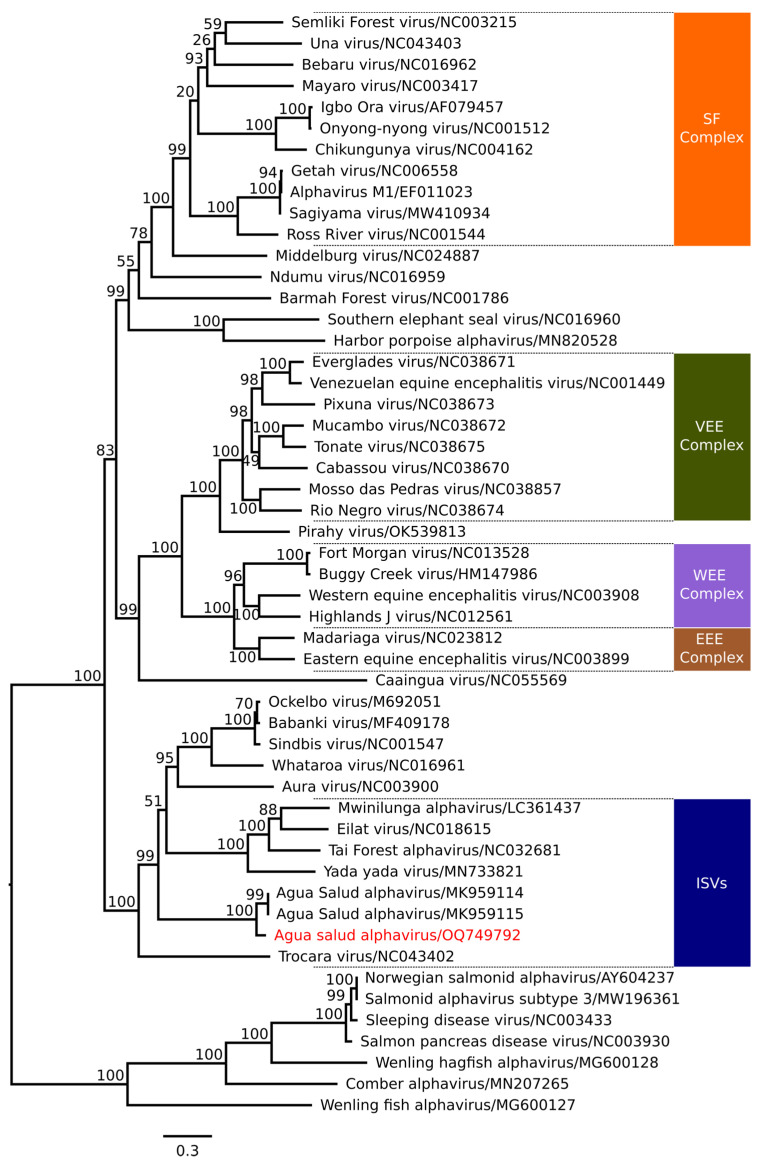
Phylogenetic relationship between the ASALV sequence isolated at the APA-Belem and complete sequences of the virus of the genus *Alphavirus* available at the NCBI. Sequence highlighted in red: ASALV isolated at the APA-Belem. SF Complex: Semliki Forest Complex. VEE Complex: Venezuelan Equine Encephalitis Complex. WEE Complex: Western Equine Encephalitis Complex. EEE Complex: Eastern Equine Encephalitis Complex. ISVs: Insect-specific alphavirus. The bootstrap values situated above branches represent the percentages derived from 1000 replicates, and the scale bar represents the nucleotide substitution rate. This phylogenetic tree was constructed based on the comparison between the intergenic regions of nucleotide sequences of structural and non-structural proteins of viruses from the genus *Alphavirus*, excluding the 5′ and 3′ regions.

Phylogenetic analyses revealed that the ASALV sequence isolated from samples collected at the APA-Belem forms a monophyletic clade with ASALV sequences isolated in Panama (GenBank Id. MK959114 and MK959115), with 100% bootstrap, representing a separate subclade composed only of the strain isolated at the APA-Belem (Figure 3). Furthermore, the ASALV sequence isolated at the APA-Belem also constitutes a basal monophyletic clade alongside the clade containing the other ISA and the clade of arboviruses infecting vertebrates, such as the *Sindbis virus* (SINV), AURAV, and *Whataroa virus* (WHAV) (51% bootstrap), forming a sister branch with a *Trocara virus* strain isolated in the Brazilian Amazon in 1984 (GenBank Id. NC_043402), with 99% bootstrap.

The alignment between the ORFs of the ASALV isolate sequence from APA-Belem and the ASALV sequences isolated in Panama in 2013 (Supplementary Figures S1–S3) demonstrated the presence of nucleotide mutations in the ASALV isolate sequence from APA-Belem, but with a high amino acid similarity between them (approximately 97%) and approximately 91% nucleotide similarity.

**Figure 4:**
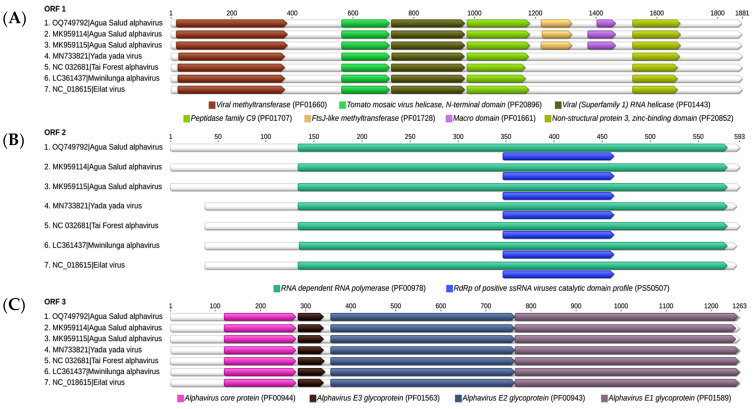
Alignment between the ORFs of ASALV sequence OQ749792 and the ORFs of insect-specific alphaviruses sequences available on NCBI, demonstrating their functional domains and the main proteins encoded by them. (**A**) Insect-specific alphavirus non-structural polyprotein nsP 1-3; (**B**) Insect-specific alphavirus non-structural polyprotein nsP4; (**C**) Insect-specific alphavirus structural polyprotein.

To better demonstrate the relationship between the ASALV strain (Brazilian and Panamanians) and the other insect-specific alphavirus, we aligned the sequences of these viruses available at NCBI and analyzed the functional domains (Figure 4). Our results showed that the ASALV sequence strain isolated at APA-Belem shares the same functional domains that encode structural proteins and non-structural proteins of other alphaviruses that form the clade of insect-specific alphaviruses, except the *FtsJ-like methyltransferase domain* (PF01728) present in the nsP2 domain, which is common only among the sequences of *Agua Salud alphavirus*.

## 4. Discussion

The *Agua Salud alphavirus* categorized as an insect-specific alphavirus (ISA) was first isolated in Panama in 2013. It was initially discovered in *Culex* (*Culex*) *declarator* mosquitoes collected in a forest fragment adjacent to rural areas [17]. However, since then, no further isolation of this virus from mosquito samples collected in the field has been reported. The present study reported an ASALV strain isolated from a batch of mosquitoes of the subgenus *Culex* (*Melanoconion*) collected in a fragment of secondary forest at the APA-Belem. This is the first report of ASALV detection within mosquitoes collected from the Brazilian Amazon.

Similar to other insect-specific alphavirus strains, including the ASALV, described by Hermanns et al. [14,17] and Torii et al. [15], the ASALV strain described in the present study was isolated from mosquitoes of the genus *Culex* sp. collected in a fragment of tropical forest close to areas with human activity. Four of the five insect-specific alphavirus (EILV, MWAV, TALV, and ASALV) have been previously isolated from *Culex* sp. mosquitoes [14,15,17,29], suggesting that insects of this genus are natural hosts for these viruses.

Some studies suggest an evolutionary relationship between arboviruses and ISVs, as seen with insect-specific alphaviruses that exhibit a basal relationship with alphaviruses comprising the western equine encephalitis complex, suggesting that these ISVs may be ancestors of these vertebrate–pathogenic viruses [12,13,14,30]. The enhancement of viral infectivity, associated with the absence of adaptive immunity, increases the tolerance of insects to these infectious agents, ensuring greater viral adaptation [31]. In this context, mosquitoes play a central role in the replication cycle of arboviruses and ISVs, acting as permanent hosts. Once infected, these insects can remain so for their entire lives, serving as continuous reservoirs of infection [32].

Adult females of the genus *Culex* sp. have low specificity and can feed on the blood of fish, amphibians, reptiles, birds, and mammals, including humans. Mosquitoes belonging to the subgenus *Culex* (*Melanoconion*) are hematophagous insects known for their preference for animal blood. They typically exhibit crepuscular habits and possess a moderate capacity to adapt to environments modified by human activities [33]. The ASALV strain found in the present study was isolated from a batch of *Culex* (*Melanoconion*) mosquitoes collected in a remnant secondary forest constantly impacted by anthropogenic actions, due to being located within an extensive urban area. Environmental changes associated with deforestation and unplanned urban growth exert significant environmental pressure in populations of vectors and animals, bringing it closer to humans [4,34].

This narrowing of the vector–host relationship favors the rapid spread of viruses among species of invertebrates and vertebrates, enabling the occurrence of viral co-infection and events of recombination, reassortment, and mutations between different viruses within the vector insects [35,36], potentially leading to the emergence of new viruses with relevance to One Health, as has occurred with WEEV and *Hightlands J virus* (HJV), which are descendants of recombination events between *Sindbis-like virus* and EEEV [37,38].

Although isolated from mosquitoes of the subgenus *Culex* (*Melanoconion*) and exhibiting phylogenetic relatedness to alphaviruses affecting vertebrates, the ASALV strain described in this study failed to elicit discernible changes in the Vero cell’s morphology indicative of viral replication when inoculated, which was also negative in the IFF test. Our results agree with those of Hermann et al. [17] which demonstrated that ASALV cannot replicate in cell lines from mammals (Vero, BHK-21, and HEK 293), reptiles (VH2), amphibians (ICR-2A), fish (BF-2, CHSE-214, and FMH), or in vivo in newborn mice, due to a non-ideal temperature range for viral replication (temperatures below 31 °C) and mismatched interaction between viral RNA and cofactors present in the vertebrates’ cells. However, the sample BE AR 867257 caused a cytopathic effect (CPE) in C6/36 cells. We did not describe here the CPE observed because the batch of mosquitoes that composed the sample BE AR 867257 was also infected by two other ISVs of the taxon *Negevirus* (*Loreto virus* and *Terra Firme virus*; unpublished data); as such, we were not able to distinguish the CPE of each one. Kallies et al. [39] reported that negeviruses are insect-specific viruses that cause high-viral-load infections, present rapid growth in mosquito cell lines, and produce moderate-to-severe cytopathic effects in C6/36 cell cultures. Thus, the presence of two negeviruses in the same batch of mosquitoes where the ASALV strain from APA-Belem was isolated made it difficult to interpret the cytopathic effect caused by this insect-specific alphavirus, suggesting the need for further studies involving the purification of the BE AR 867257 sample and the separation of the viruses present in it.

During the phylogenetic analysis, it was observed that the ASALV sequence isolated in APA-Belem forms a monophyletic clade with the ASALV sequences isolated in Panama in 2013, with 100% bootstrap support. Additionally, the identity matrix comparing the nucleotide and amino acid similarity percentages between the coding ORFs for structural and non-structural proteins of the ASALV sequence isolated in APA-Belem and the ORFs of insect-specific alphavirus sequences deposited in the NCBI database demonstrated that the sequence of the ASALV isolate from APA-Belem shows high nucleotide and amino acid similarity with the ASALV strains isolated in Panama, suggesting that the APA-Belem isolate represents a distinct ASALV strain from the Panamanian strains. According to Chen et al. [40], members of most *Alphavirus* species exhibit at least 10% amino acid identity divergence in their coding regions; however, this is not the only parameter that should be considered when classifying a virus within the same species. In addition to the low percentage of amino acid divergence, to be considered a strain of an *Alphavirus* species, the virus in question must also exhibit antigenic and ecological characteristics in common with other members of the species, share the same vectors and hosts, and cause similar cellular changes and symptoms during the infection [40]. Although isolated in different countries, the ASALV strain isolated in APA-Belem shares many characteristics in common with the Panamanian ASALV strains, among them the host of the same genus (*Culex* sp.), the inability to replicate in vitro in cells of vertebrates, and genetic characteristics such as functional domains and structural and non-structural proteins produced by them.

The alignment between the ORFs of the ASALV isolate sequence from APA-Belem and the ORFs of insect-specific alphavirus sequences deposited in NCBI showed that the sequence isolated in APA-Belem shares the same functional domains as the sequences of other insect-specific alphaviruses, except the *FtsJ-like methyltransferase* domain associated with the nsP2 domain, which is common only among the *Agua Salud alphavirus* sequences. In alphaviruses, the *FtsJ-like methyltransferase* domain represents a 5′ cap-0 structure, which is present in mRNA and promotes stability and protection of the genetic material against cellular recognition and activation of the host immune system [41]. In pathogenic alphaviruses for vertebrates such as CHIKV, VEEV, and SINV, this cap is capable of causing host cell transcription shutdown, reducing interferon expression, and promoting viral replication [42,43]. However, the function of the *FtsJ-like methyltransferase* domain in the ASALV replication cycle, or its influence on the host’s immune system, is poorly elucidated.

Although authors such as Powers et al. [44] and Hermanns et al. [14] suggest that the fact that interactions between alphaviruses and their hosts are specific, and the low prevalence of insect-specific alphaviruses in mosquito populations may be geographic restriction factors for these viruses; the detection of a strain of *Agua Salud alphavirus* in mosquitoes collected at APA-Belem indicates the circulation of this alphavirus in the population of hematophagous arthropods that inhabit forest fragments in the Amazon biome.

Renowned for its unparalleled richness in animal and plant biodiversity, the Brazilian Amazon biome harbors a plethora of insect species and climate conditions favorable to the reproduction of hematophagous arthropods vectoring arboviruses and other viruses with medical importance [45]. The surveillance actions including hematophagous arthropod collection and molecular diagnostic tools have considerably boosted the discovery and characterization of new viruses in the Amazon region, including new ISVs [6,7,8] such as the described in this study.

The identification of novel ISV strains has great scientific implications for global One Health, as these viruses have a potential application in combating arbovirus vectors as well as a possible applicability in the production of safe vaccines for the major pathogenic arboviruses affecting both humans and animals [12,46,47]. Nasar et al. [47] reported that in vitro and in vivo EILV infection can negatively interfere with the replication dynamics of alphaviruses pathogenic to humans and animals, such as SINV, VEEV, EEEV, WEEV, and CHIKV, reducing the viral load and delaying the replication period in both C7/10 cells and the midgut of *Aedes* sp. mosquitoes.

Some studies have shown that *Agua Salud alphavirus* can interfere with the replication dynamics of pathogenic arboviruses important for One Health, both in vitro and in vivo. These studies highlight ASALV’s ability to induce antiviral pathways through miRNA production in the intestinal tract of arbovirus vector mosquitoes such as *Aedes* aegypti [48,49]. Furthermore, Jagtap et al. [50] have demonstrated ASALV’s high tropism for central nervous system cells of *Ae. aegypti*, suggesting that this virus may negatively interfere with the feeding behavior of these mosquitoes, reducing their capacity to search for the blood of vertebrate hosts and reducing the risk of arbovirus transmission.

## 5. Conclusions

In summary, this study marks a significant milestone as the report of insect-specific alphavirus isolation within Brazil, alongside providing the first description of *Agua Salud* alphavirus isolation within the Brazilian Amazon Forest. The ASALV strain isolated in APA-Belem aligns with the existing literature, having been isolated from samples of mosquitos’ *Culex* sp. and exhibiting in vitro replication only within insect cells.

Currently, environmental changes are leading to the emergence of endemic diseases in the Amazon region. The use of ISVs for controlling mosquito vectors responsible for transmitting viruses capable of causing human and animal diseases is an attractive prospect for improving human and animal health. Thus, the results of this study highlight the importance of further investigations into virus circulation relevant to One Health in insect vectors from samples collected from urban areas and the Brazilian Amazon.

## Figures and Tables

**Table 1:** Polyclonal antibodies for antigenic groups of arboviruses used in the IIF test to detect viral presence in C6/36 and Vero cell cultures inoculated with supernatant of macerated hematophagous arthropods collected in APA-Belem, Pará, Brazil.

Genus	Antigenic Group	Virus
*Alphavirus*	A	*Aura virus* (AURAV), EEEV, *Mayaro virus* (MAYV), MUCV, PIXV, *Una virus* (UNAV), WEEV, *Chikungunya virus* (CHIKV), *Trocara virus* (TROV).
*Orthoflavivirus*	B	*Orthoflavivirus denguei* (1-4), *Orthoflavivirus flavi*, *Bussuquara virus*, *Orthoflavivirus cacipacoreense*, *Orthoflavivirus ilheusenses*, *Orthoflavivirus nilense*, *Orthoflavivirus louisense*, *Orthoflavivirus zikaense.*
*Orthobunyavirus*	Guamá	*Orthobunyavirus ananindeuaense*, *Bimiti orthobunyavirus*, *Orthobunyavirus catuense*, *Orthobunyavirus guamaense*, *Orthobunyavirus mirimense*, *Moju orthobunyavirus*, *Orthobunyavirus timboteuaense.*
	Capim	*Orthobunyavirus acaraense*, *Benevides orthobunyavirus*, *Orthobunyavirus benficaense*, *Orthobunyavirus capimense*, *Orthobunyavirus guajaraense, Orthobunyavirus bushbushense, Moriche orthobunyavirus*.
	Bunyamwera	*Orthobunyavirus iacoense*, *Kairi orthobunyavirus*, *Orthobunyavirus macauaense, Orthobunyavirus maguariense*, *Sorocaba orthobunyavirus*, *Tucunduba orthobunyavirus*, *Taiassui orthobunyavirus*, *Xingu orthobunyavirus.*
	Simbu	*Orthobunyavirus oropoucheense*
*Phlebovirus*	Phlebotomus	*Phlebovirus alenquerense, Phlebovirus ambeense, Ariquemes phlebovirus, Belterra phlebovirus, Phlebovirus bujaruense, Phlebovirus candiruense*, *Phlebovirus icoaraciense, Joá phlebovirus, Phlebovirus itaporanguense, Jacundá phlebovirus, Morumbi phlebovirus, Mucura phlebovirus, Phlebovirus mugumbaense, Phlebovirus oriximinaense, Pacuí phlebovirus, Phlebovirus saloboense, Phlebovirus taparaense, Phlebovirus turunaense, Phlebovirus uriuranaense, Phlebovirus urucuriense.*
*Orbivirus*	Changuinola	*Acatinga virus, Acurené virus, Almeirim virus, Altamira virus, Anapú virus, Araçaí virus, Aratau virus, Aruana virus, Arawetê virus, Assurinis virus, Bacajaí virus, Bacuri virus, Balbina virus, Barcarena virus, Breves virus, Canindé virus, Canoal virus, Catetê virus, Orbivirus changuinolaense*, *Coari virus, Gorotire virus, Gurupi virus, Iopaka virus, Ipixaia virus, Irituia virus, Iruana virus, Itaboca virus, Jamanxi virus, Jandaia virus, Jari virus, Jatuarana virus, Jutaí virus, Kararaô virus, Melgaço virus, Monte Dourado virus, Ourém virus, Pacajá virus, Parakanã virus, Poranati virus, Parauapebas virus, Parú virus, Pependana virus, Pindobai virus, Piratuba virus, Purus virus, Rio Mutapi virus, Saracá virus, Serra Sul virus, Surubim virus, Tapiropé virus, Tekupeú virus, Timbozal virus, Tocantins virus, Tocaxá virus, Tuerê virus, Tumucumaque virus, Uatamã virus, Uxituba virus, Xaraíra virus, Xiwanga virus.*

**Table 2:** Nucleotide and amino acid identity matrix between structural proteins of ASALV isolated in APA-Belem and insect-specific alphaviruses structural proteins available in NCBI.

	Insect-Specific Alphavirus Strain	1	2	3	4	5	6	7	
1	*Eilat virus*/NC_018615		72.27	71.87	62.05	45.12	45.12	44.72	Amino acid
2	*Mwinilunga alphavirus*/LC361437	69.18		75.93	63.33	46.12	46.12	46.04	
3	*Tai Forest alphavirus*/NC_032681	66.85	68.83		62.45	46.32	46.32	46.40	
4	*Yada yada virus*/MN733821	61.55	61.88	61.62		44.56	44.56	44.24	
5	*Agua Salud alphavirus*/MK959114	53.06	52.61	53.71	53.29		91.06	96.96	
6	*Agua Salud alphavirus*/MK959115	53.03	52.58	53.71	53.27	99.97		96.96	
7	*Agua Salud alphavirus*/OQ749792	52.85	52.42	53.68	52.89	91.06	91.03		
		Nucleotide							

Sequence highlighted in red: *Agua Salud alphavirus* strain isolated in the APA-Belem. Values shaded in orange: percentage of nucleotide similarity between the *Agua Salud alphavirus* sequences. Values shaded in yellow: percentage of amino acid similarity between the *Agua Salud alphavirus* sequences. Numbers written in green: percentage of nucleotide similarity between the ASALV strain isolated in APA-Belem and the ASALV strains isolated in Panama. Numbers written in blue: percentage of amino acid similarity between the ASALV strain isolated in APA-Belem and the ASALV strains isolated in Panama.

**Table 3:** Nucleotide and amino acid identity matrix between non-structural proteins of ASALV isolated in APA-Belem and insect-specific alphavirus non-structural proteins available in NCBI.

	Insect-Specific Alphavirus Strain	1	2	3	4	5	6	7	
1	*Eilat virus* /NC_018615		83.25	81.80	75.58	62.56	62.47	62.56	Amino acid
2	*Mwinilunga alphavirus*/LC361437	72.87		80.62	74.33	62.55	62.51	62.59	
3	*Tai Forest alphavirus*/NC_032681	72.32	71.39		74.19	62.15	62.15	62.34	
4	*Yada yada virus*/MN733821	67.93	68.08	67.33		60.87	60.87	60.83	
5	*Agua Salud alphavirus*/MK959114	62.17	61.42	60.94	60.15		99.88	98.19	
6	*Agua Salud alphavirus*/MK959115	62.14	61.42	60.94	60.15	99.96		98.06	
7	*Agua Salud alphavirus*/OQ749792	62.09	61.38	60.89	60.23	89.77	89.73		
		Nucleotide							

Sequence highlighted in red: *Agua Salud alphavirus* strain isolated in the APA-Belem. Values shaded in orange: percentage of nucleotide similarity between the *Agua Salud alphavirus* sequences. Values shaded in yellow: percentage of amino acid similarity between the *Agua Salud alphavirus* sequences. Numbers written in green: percentage of nucleotide similarity between the ASALV strain isolated in APA-Belem and the ASALV strains isolated in Panama. Numbers written in blue: percentage of amino acid similarity between the ASALV strain isolated in APA-Belem and the ASALV strains isolated in Panama.

## Data Availability

Not applicable. Data are contained within the article and supplementary materials.

## Supplementary Materials

The following supporting information can be downloaded at https://www.mdpi.com/article/10.3390/v16091355/s1, Figure S1: Alignment between the nsP1-3 non-structural protein functional domain (ORF1) of the *Agua Salud alphavirus* sequence isolated in APA-Belem (OQ749792) and the nsP1-3 non-structural protein functional domain of ASALV sequences available in the NCBI, demonstrating the nucleotide and amino acid mutation points of the OQ749792 sequence in relation to ASALV strains isolated in Panama in 2013; Figure S2: Alignment between the nsP4 non-structural functional domain (ORF2) of the *Agua Salud alphavirus* sequence isolated in APA-Belem (OQ749792) and the nsP4 non-structural functional domain of ASALV sequences available in the NCBI, demonstrating the nucleotide and amino acid mutation points of the OQ749792 sequence in relation to ASALV strains isolated in Panama in 2013; Figure S3: Alignment between the structural protein functional domain (ORF3) of the *Agua Salud alphavirus* sequence isolated in APA-Belem (OQ749792) and the structural protein functional domain of ASALV sequences available in the NCBI, demonstrating the nucleotide and amino acid mutation points of the OQ749792 sequence in relation to ASALV strains isolated in Panama in 2013.
