# Supplementary figures and images for: The First Isolation of Insect-Specific Alphavirus (Agua Salud alphavirus) in Culex (Melanoconion) Mosquitoes in the Brazilian Amazon
Source: Viruses. 2024 Aug 24;16(9):1355. doi: 10.3390/v16091355 (PMC11436152; doi:10.3390/v16091355)

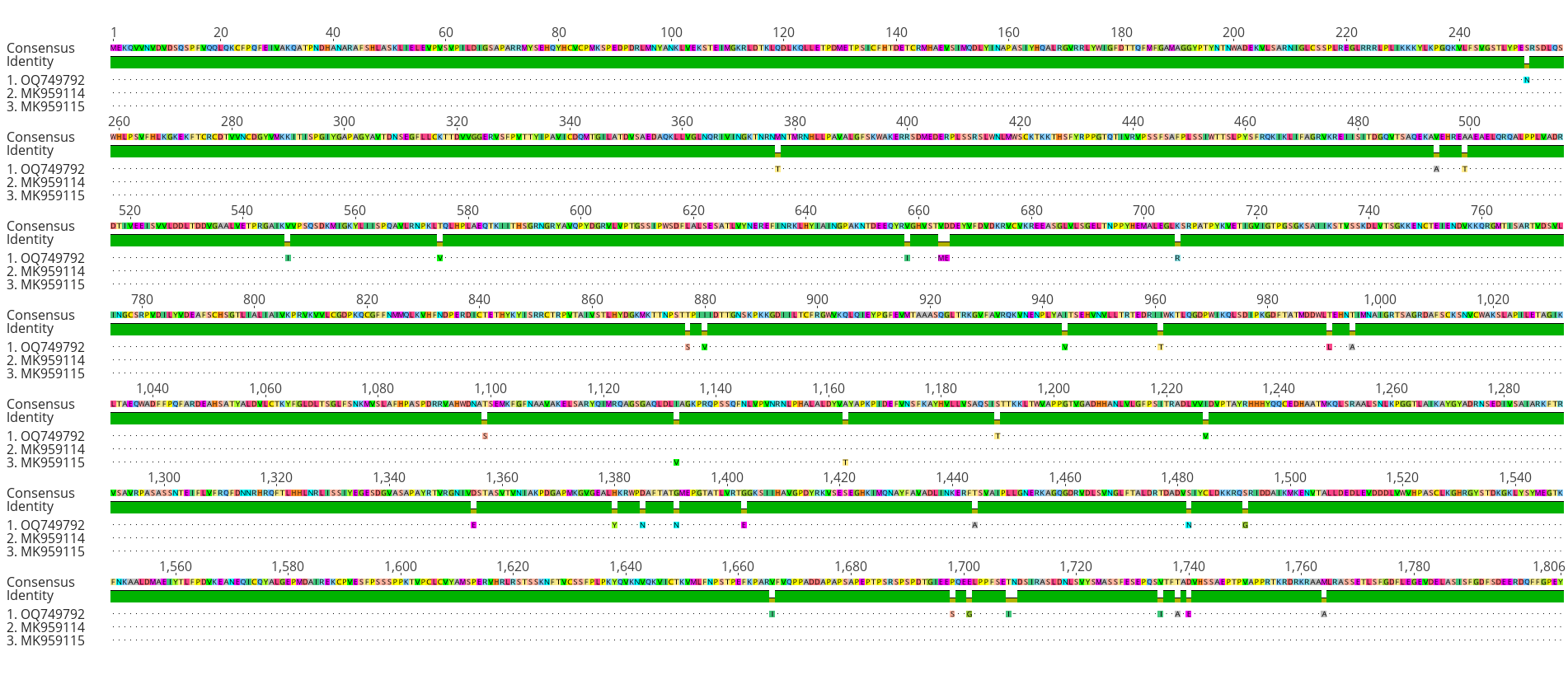

Supplement: Supplementary file 1 [file viruses-16-01355-s001.zip › Figure-S1- tiff 300DPI.tiff]

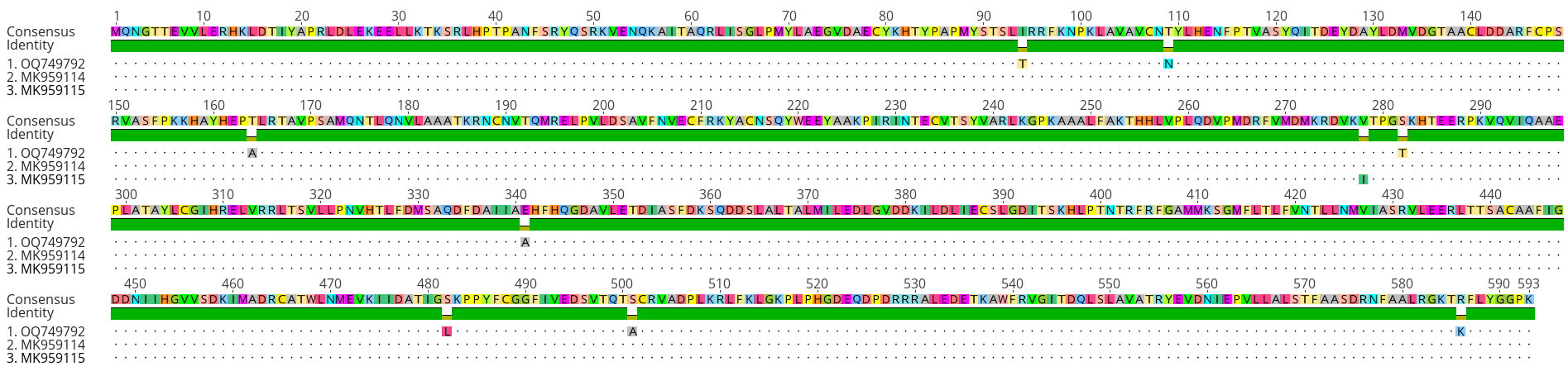

Supplement: Supplementary file 1 [file viruses-16-01355-s001.zip › Figure-S2 - tiff 300DPI.tiff]

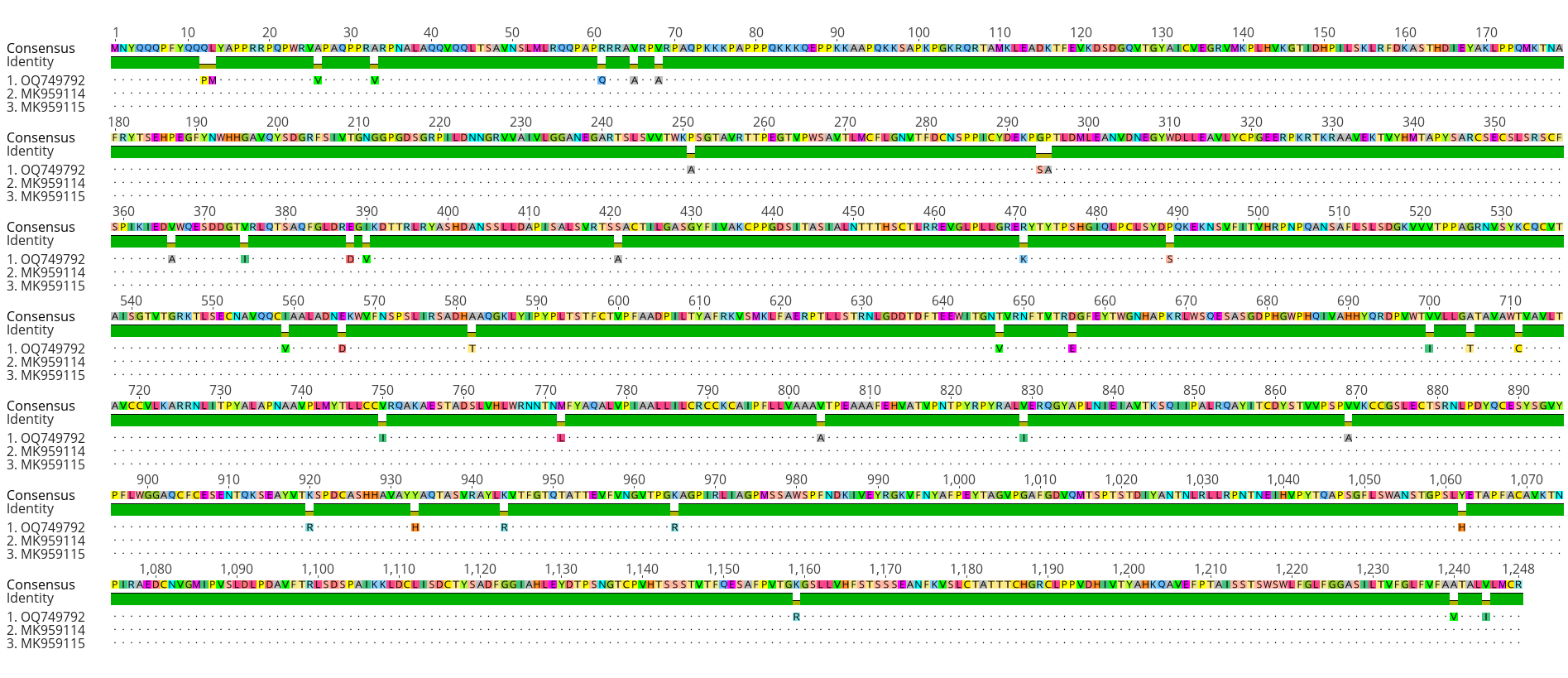

Supplement: Supplementary file 1 [file viruses-16-01355-s001.zip › Figure-S3- tiff 300DPI.tiff]
